# Nontuberculous Mycobacteria Infections at a Provincial Reference Hospital, Cambodia

**DOI:** 10.3201/eid2307.170060

**Published:** 2017-07

**Authors:** Maryline Bonnet, Kim Chamroeun San, Yati Pho, Chandara Sok, Jean-Philippe Dousset, William Brant, Northan Hurtado, Khun Kim Eam, Elisa Ardizzoni, Seiha Heng, Sylvain Godreuil, Wing-Wai Yew, Cathy Hewison

**Affiliations:** TRANSVIHMI IRD, INSERM, University of Montpellier, Montpellier, France (M. Bonnet);; Epicentre, Paris, France (M. Bonnet, Y. Pho);; Médecins Sans Frontières, Paris (K. Chamroeun San, C. Sok, J.-P. Dousset, N. Hurtado, E. Ardizzoni, C. Hewison);; University of Virginia, Charlottesville, Virginia, USA (W. Brant);; National Center for Tuberculosis and Leprosy Control, Phnom Penh, Cambodia (K.-K. Eam);; Institute of Tropical Medicine, Antwerp, Belgium (E. Ardizzoni);; Institut Pasteur, Phnom Penh (S. Heng);; INSERM, University of Montpellier, Montpellier (S. Godreuil);; Chinese University of Hong Kong, Hong Kong, China (W.-W. Yew)

**Keywords:** nontuberculous mycobacteria, NTM, tuberculosis and other mycobacteria, TB, limited-resource countries, infectious diseases, Cambodia, bacteria

## Abstract

Prevalence of nontuberculous mycobacteria (NTM) disease is poorly documented in countries with high prevalence of tuberculosis (TB). We describe prevalence, risk factors, and TB program implications for NTM isolates and disease in Cambodia. A prospective cohort of 1,183 patients with presumptive TB underwent epidemiologic, clinical, radiologic, and microbiologic evaluation, including >12-months of follow-up for patients with NTM isolates. Prevalence of NTM isolates was 10.8% and of disease was 0.9%; 217 (18.3%) patients had TB. Of 197 smear-positive patients, 171 (86.8%) had TB confirmed (167 by culture and 4 by Xpert MTB/RIF assay only) and 11 (5.6%) had NTM isolates. HIV infection and past TB were independently associated with having NTM isolates. Improved detection of NTM isolates in Cambodia might require more systematic use of mycobacterial culture and the use of Xpert MTB/RIF to confirm smear-positive TB cases, especially in patients with HIV infection or a history of TB.

Nontuberculous mycobacteria (NTM) include ≈160 species of environmental mycobacteria found largely in soil and water sources, of which ≈40 can be associated with lung disease, the most common clinical presentation of NTM infection (*1*,*2*). NTM are usually less pathogenic than *Mycobacterium tuberculosis* complex (MTBc) and can be isolated without the presence of disease (colonization) (*3*). Pulmonary disease attributable to NTM infection frequently occurs in patients with structural lung disease (*1*,*4*). In HIV-infected patients, disseminated NTM disease occurs more frequently once the CD4 cell count is <50 cells/μL (*5*).

NTM infections are not reportable diseases, and most available data come from sentinel surveillance and laboratory-based studies; consequently, the exact prevalence of such infections is not well known (*6*,*7*). However, detection of NTM isolates has increased worldwide, a trend that might be attributed to several factors, including a surge of HIV infections in the past 2 decades, a better understanding of the clinical and pathological relationship between host and pathogen, improved detection methods, increased natural or artificial environmental exposure (i.e., tap water in developing countries), population aging, and improved survival of patients with structural lung diseases (*8*–*15*). An increase of NTM isolates was also observed after the introduction of liquid culture methods, which are more sensitive in detecting NTM isolates than solid methods (*14*). However, because mycobacterial culture laboratories are lacking in limited-resource countries, the burden of NTM infection is often poorly documented in areas with a high tuberculosis (TB) prevalence. This observation is particularly true in Asia, where no population-based studies have been conducted to document the epidemiology of NTM pulmonary isolates and NTM lung disease (*4*).

Some studies indicate that NTM might play an important role in TB-like disease, which can lead to inappropriate or unnecessary anti-TB treatment (*15*–*18*). Furthermore, smear microscopy cannot distinguish MTBc from NTM, and in many countries with high TB prevalence, access to the XpertMTB/RIF assay (Cepheid, Sunnyvale, CA, USA) is still limited (*19*). Therefore, the effect of NTM isolates on TB case management needs to be further explored in countries with high TB prevalence.

Current guidelines for diagnosis and treatment of NTM infection rely mainly on criteria established by the American Thoracic Society (ATS) in 2007 (*1*,*15*,*20*). Despite their usefulness, the ATS criteria were principally designed for diagnosis of lung disease caused by *M. kansasii*, *M. abscessus*, and *M. avium* complex (comprising *M. avium* and *M. intracellulare*), all of which are common in North America and possibly less adapted to the ecologic situation of other regions in the world (*1*,*15*,*21*–*24*). These criteria are less appropriate for the diagnosis of disseminated NTM infection occurring in immunocompromised patients and can require the use of computed tomography scan and advanced diagnostic technologies that are not available in many resource-limited countries (*20*).

The primary objective of our study was to estimate the prevalence of NTM isolates and NTM lung disease among consecutive patients with presumptive TB at the chest clinic of the Kampong Cham Provincial Reference Hospital in Cambodia. Secondary objectives were to identify factors associated with isolation of NTM and to compare the epidemiologic, clinical, and radiologic findings between patients diagnosed with TB, NTM lung disease, and NTM colonization. Finally, exploratory objectives aimed to review the use of the ATS criteria for diagnosis of NTM lung disease and to assess the effect of NTM isolates on TB case management in a setting with high TB prevalence but limited diagnostic capacity.

## Materials and Methods

### Study Design and Population

The study involved a prospective cohort of patients (>15 years of age) with presumptive pulmonary TB (cough for >3 weeks), ability to produce 2 sputum specimens, and no anti-TB treatment for >7 days during the previous month. The reference hospital received support from Médecins Sans Frontières for diagnosis and treatment of TB for a population of ≈300,000 inhabitants in the Kampong Cham province.

### Procedures

At enrollment, patients were interviewed about potential risk factors for NTM exposure and NTM lung disease and underwent a physical examination. HIV testing in accordance with national guidelines and postero-anterior chest radiographs were performed. Three sputum specimens collected during 2 consecutive days were examined by using LED-fluorescent microscopy after auramine-O staining. The 2 best specimens based on macroscopic appearance (purulent and mucopurulent specimens) were decontaminated by using the N-acetyl-L-cysteine-sodium hydroxide method (2% final concentration and 20 min digestion) and then centrifuged. The sediment from the first sample was cultured by using the BBL Mycobacteria Growth Indicator Tube (MGIT) manual system (Becton Dickinson, Sparks, MD, USA) and the second by using Lowenstein-Jensen (LJ) media (*25*). LJ cultures were read once per week for 8 weeks and MGIT cultures once a day for 56 days. Negative culture results were delivered after 8 weeks. The third specimen was stored at −20°C and cultured on LJ and MGIT if NTM was isolated on only 1 of the 2 initial cultures to increase the possibility of isolating the same NTM species in 2 different specimens or in the event 1 of the 2 cultures was contaminated. Identification of MTBc or NTM species used the P-nitrobenzoic acid and Bioline Ag MPT64 Rapid (Standard Diagnostics Inc., Kyonggi-do, Korea) tests. In case of NTM growth on any of the 2 cultures, a subculture on LJ was sent to the Institut Pasteur in Phnom Penh for rapid NTM speciation using 2 DNA strip assays (GenoType Mycobacterium CM and GenoType Mycobacterium AS, Lifescience, Nehren, Germany). An XpertMTB/RIF assay was performed on smear-positive samples according to the manufacturer’s guidelines. To reduce risk for specimen contamination with environmental mycobacteria, samples were collected after rinsing the mouth with mineral water and laboratory procedures were carried out with filtered water.

### Case Definitions, Treatment, and Follow-up

In the absence of universal case definitions, patients with culture-positive NTM were classified as having NTM lung disease or NTM colonization by a study expert committee based on review of microbiologic, clinical, and radiologic information at baseline and during follow-up (*15*). The definition of NTM lung disease was adapted from the 2007 ATS criteria requiring pulmonary symptoms and abnormal chest radiograph suggestive of TB or NTM disease (i.e., infiltrates, nodular, or cavitary opacities); growth of the same NTM species from >2 sputum samples collected at different times; and exclusion of other differential diagnoses, such as TB (*1*). Patients who had NTM isolates but did not meet the eligible criteria for NTM lung disease were classified as having NTM colonization. The decision to initiate treatment was guided by the study expert committee based on the potential risks and benefits of a prolonged course of multiple antibiotics for the patient, taking into consideration age, comorbid conditions, and disease type (*1*,*4*,*11*).

Treatment regimens were based on the 2007 ATS guidelines, and patients were followed until 12 months after completion of treatment with monthly sputum smear microscopy and culture (*1*). Patients classified as having NTM colonization were followed for 12 months. Among these patients, those who remained symptomatic at the first follow-up visit for culture results (2–8 weeks after enrollment) were classified having as symptomatic NTM colonization and were followed every 3 months. Asymptomatic patients or patients with substantial clinical improvement were classified as having asymptomatic NTM colonization and were followed at 6 and 12 months only. Sputum smear microscopy and culture and chest radiograph during follow-up visits were only performed in patients who were still symptomatic. Patients with both MTBc and NTM isolates were classified as having TB. Diagnosis of NTM lung disease could be reconsidered if patients did not respond to TB treatment.

### Sample Size and Statistics

By using an NTM disease prevalence estimation of 7% among patients with presumptive TB, a precision of 1.5%, a risk α of 5%, and 10% increase for drop-outs, we determined that a sample size of 1,222 patients was needed (*26*). Data were double entered by using Epi-Data 3.0 software (EpiData Association, Odense Denmark) and analyzed in Stata 10 software (StataCorp LP, College Station, TX, USA). We calculated 95% CIs for prevalences of NTM isolates and NTM lung disease. Patients’ characteristics at enrollment were displayed according to the final patients’ classification (i.e., as having TB, NTM lung disease, or NTM colonization, or being culture-negative). We performed univariate and multivariate regression analysis using logistic regression model to assess the association between baseline patient characteristics and the isolation of NTM. We included covariates associated with a p value <0.4 in univariate analysis in the initial multivariate model and used a manual backward stepwise approach to obtain the final multivariate model. Statistical significance (p<0.05) was assessed with the likelihood-ratio test. The proportion of MTBc isolates detected using the XpertMTB/RIF assay was calculated for smear-positive patients. Criteria used for the diagnosis of NTM lung disease were compared with the 2007 ATS criteria for patients with >1 NTM isolate.

### Ethics

The study protocol was approved by the National Ethics Committee for Health Research, Phnom Penh, Cambodia, and the Comité de Protection des Personnes, Saint Germain en Laye, France. Patients’ informed consent was obtained.

## Results

A total of 1,183 patients with laboratory culture results were enrolled during October 1, 2012–April 21, 2014 (Figure). The prevalence of NTM isolates (including those detected in 4 patients with NTM and MTBc isolates) was 10.8% (95% CI 9.1%–12.7%); prevalence of NTM lung disease was 0.9% (95% CI 0.5%–1.7%). Of the 113 patients with NTM colonization, 61 (54.0%) were still symptomatic at the time of culture results. Of the 124 patients classified as having NTM lung disease or NTM colonization, 24 (19.3%) were lost to follow-up, 9 (7.3%) withdrew voluntarily, and 9 (7.3%) died.

**Figure:**
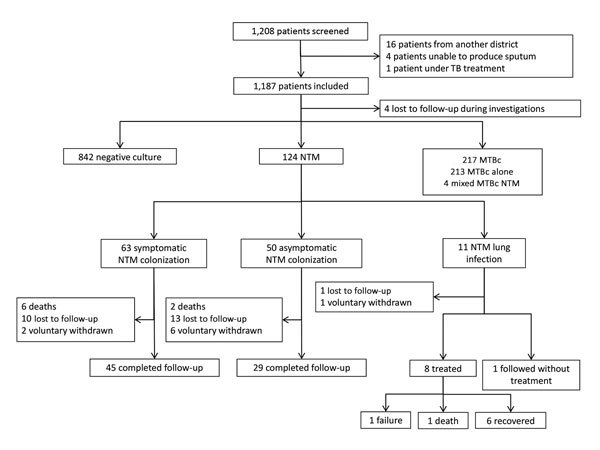
Schematic summary of results from study of NTM infections at Kampong Cham Provincial Reference Hospital, Cambodia, October 1, 2012–April 21, 2014. MTBc, *Mycobacterium tuberculosis* complex; NTM, nontuberculous mycobacteria; TB, tuberculosis.

The male:female ratio among enrollees was 0.94, and median age was 54 years. Almost half of patients were farmers, and 26.0% had a TB history (median time since the last event 3 years, interquartile range 2–8 years). More than one third of patients had chronic bronchitis, and 12.1% had been hospitalized in the past for respiratory disease (Table 1). TB history (odds ratio [OR] 29.75, 95% CI 7.64–115.85), HIV infection (OR 50.57, 95% CI 7.89–324.16), and previous hospitalizations for respiratory disease (OR 17.4, 95% CI 4.70–64.14) were associated with a diagnosis of NTM lung disease compared with TB. Similarly, among patients with NTM isolates, those with TB history (OR 3.59, 95% CI 0.99–13.05), HIV infection (OR 23.43, 95% CI 3.63–151.19), and previous hospitalizations for respiratory disease (OR 7.84, 95% CI 2.12–28.92) were more likely to be diagnosed with NTM lung disease.

**Table 1:** Demographic and epidemiologic characteristics of patients in a study of NTM infections at Kampong Cham Provincial Reference Hospital, Cambodia, October 1, 2012–April 21, 2014*

Characteristic	Total, N = 1,187	TB, n = 217	NTM disease, n = 11	NTM colonization, n = 113	Culture-negative, n = 842
Median age, y (IQR)	54 (40–65)	49 (35–62)	54 (46–63)	57 (44–65)	54 (40–65)
Sex					
F	611 (51.5)	87 (40.1)	6 (54.5)	66 (59.5)	451 (53.6)
M	567 (48.5)	130 (60.0)	5 (45.5)	47 (41.6)	391 (46.4)
Occupation					
Unemployed	339 (28.6)	61 (28.1)	4 (36.4)	35 (31.0)	238 (28.3)
Student	35 (2.9)	7 (3.2)	0	5 (4.4)	23 (2.7)
Pensioner	25 (2.1)	1 (0.5)	0	4 (3.5)	20 (2.4)
Farmer	568 (47.9)	109 (50.2)	7 (63.6)	45 (39.8)	406 (48.2)
Trader/seller	57 (4.8)	7 (3.2)	0	8 (7.1)	42 (5.0)
Building worker	18 (1.5)	5 (2.3)	0	1 (0.9)	12 (1.4)
Police/army	14 (1.2)	5 (2.3)	0	0	8 (0.9)
Fisherman	19 (1.6)	2 (0.9)	0	4 (3.5)	13 (1.5)
Administration	33 (2.8)	3 (1.4)	0	4 (3.5)	26 (3.1)
Factory worker	45 (3.8)	13 (6.0)	0	2 (1.8)	30 (3.6)
Other	34 (2.9)	4 (1.8)	0	5 (4.4)	24 (2.8)
Drinking water					
Water well	709 (59.7)	127 (58.5)	7 (63,6)	63 (55.7)	510 (60.6)
Running water	210 (17.7)	36 (16.6)	2 (18.2)	27 (23.9)	144 (17.1)
Rain	64 (5.4)	15 (6.9)	0	7 (6.2)	42 (5.0)
River/lake/pond	131 (11.0)	24 (11.1)	1 (9.1)	11 (9.7)	95 (11.3)
Mineral water	74 (6.1)	15 (6.9)	1 (9.1)	5 (4.4)	51 (6.1)
Water disinfection					
Boiled	701 (59.1)	119 (54.8)	5 (45.5)	68 (60.2)	506 (60.2)
Filtered	121 (10.2)	15 (6.9)	2 (18.2)	13 (11.5)	91 (10.8)
None	364 (30.7)	83 (38.2)	4 (36.4)	32 (28.3)	244 (29.0)
Use of hot tub	13/345 (3.8)	1/63 (1.6)	0	1/37 (2.7)	11/242 (4.5)
Pneumoconiosis risk	27 (2.3)	11 (5.1)	0	1 (0.9)	15 (1.8)
Surgery of the lung	2 (0.2)	0	0	0	2 (0.2)
Past TB	309 (26.0)	12 (5.5)	7 (63.4)	37 (32.7)	252 (30.0)
Chronic bronchitis	447/1,168 (38.3)	99/213 (46.5)	4 (36.4)	34/110 (30.6)	310/830 (33.0)
Hospitalization†	144 (12.1)	14 (6.4)	6 (54.5)	15 (13.3)	109 (13.0)
Smoking					
Current smoker	175 (14.7)	39 (17.9)	2 (18.2)	10 (8.8)	123 (14.6)
Former smoker	266 (22.4)	57 (26.3)	3 (27.3)	23 (20.3)	58 (6.9)
Esophageal motility disorders	1/1,178 (<0.1)	0	0	0	1/836 (<0.1)
HIV positive	19/917 (2.1)	2/179 (1.1)	4 (36.4)	2/84 (2.4)	11/639 (1.7)

*Values are no. patients/no. with available data (%) except as indicated. IQR, interquartile range; NTM, nontuberculous mycobacteria; TB, tuberculosis. †For respiratory disease.

A low body mass index (i.e., <18.5 kg/m^2^) was the only clinical parameter that was significantly different between patients with NTM lung disease and TB patients (90.1% vs. 56.7%; p = 0.025) (Table 2). On chest radiograph, fibrosis or volume loss (p = 0.031) and bronchiectasis (p = 0.001) were more common in patients with NTM lung disease than in TB patients. Cavitation (p = 0.029), pleural thickening (p = 0.039), and bronchiectasis (p = 0.005) were more frequent in patients with NTM lung disease than in patients with NTM colonization (Table 3). 

**Table 2:** Clinical presentation of patients in a study of NTM infections at Kampong Cham Provincial Reference Hospital, Cambodia, October 1, 2012–April 21, 2014*

Characteristic	Total, N = 1,187	TB, n = 217	NTM disease, n = 11	NTM colonization, n = 113	Culture-negative, n = 842
Temperature >37.5°C	115 (9.7)	32 (14.7)	2 (18.2)	9 (8.0)	72 (8.5)
Median BMI, kg/m^2^ (IQR)	19.0 (17.0–21.5)	18.1 (16.2–19.8)	14.8 (13.9–16.2)	18.8 (16.9–20.9)	19.4 (17.2–22.1)
BMI <18.5	524 (44.1)	123 (56.7)	10 (90.9)	52 (46.0)	337 (40.0)
Karnofsky, % (IQR)	90 (90–100)	90 (80–100)	80 (80–90)	90 (90–100)	90 (90–100)
SaO_2_, % (IQR)	98 (97–99)	98 (97–99)	99 (91–100)	98 (97–99)	98 (97–99)
Cough	1,185 (99.8)	217 (100)	11 (100)	113 (100)	840 (99.8)
Duration, wks (IQR)	4 (3–12)	8 (4–12)	8 (2–20)	4 (3–10)	4 (3–12)
Chest pain	501 (42.2)	106 (48.8)	7 (63.6)	45 (39.8)	343 (40.6)
Hemoptysis	151 (12.7)	27 (12.4)	0	16 (14.2)	108 (12.8)
Dyspnea	563 (47.4)	118 (54.4)	7 (63.6)	46 (40.7)	392 (46.6)
Loss of appetite	300 (25.3)	63 (29.0)	5 (45.4)	27 (23.9)	205 (24.3)
Loss of weight	753 (63.4)	182 (83.9)	9 (81.8)	62 (54.9)	499 (59.3)
Fatigue	151 (12.7)	44 (20.3)	4 (36.7)	10 (8.8)	93 (11.0)
Pleural effusion	18 (1.5)	3 (1.4)	0	1 (0.9)	14 (1.7)
Adenopathy	5 (0.4)	1 (0.5)	0	0	4 (0.5)

*Values are no. (%) patients except as indicated. IQR, interquartile range; NTM, nontuberculous mycobacteria; TB, tuberculosis.

**Table 3:** Radiologic presentation of patients in a study of NTM infections at Kampong Cham Provincial Reference Hospital, Cambodia, October 1, 2012–April 21, 2014*

Characteristic	No. (%) patients				
	Total, N = 1,187	TB, n = 217	NTM disease, n = 11	NTM colonization, n = 113	Culture-negative, n = 842
Normal	260 (21.9)	2 (<0.1)	1 (9.1)	44 (38.9)	212 (25.2)
Abnormal	927 (78.1)	215 (99.1)	10 (91.0)	69 (61.1)	630 (74.8)
Cavity	189 (20.4)	113 (52.6)	4 (40.0)	7 (10.1)	64 (10.2)
Fibrosis/volume loss	335 (36.2)	76 (35.5)	7 (70.0)	27 (39.1)	224 (35.6)
Infiltrates	866 (93.4)	214 (99.5)	10 (100)	63 (91.3)	577 (91.6)
Miliary	8 (0.9)	8 (3.7)	0	0	0
Adenopathy	103 (11.1)	24 (11.2)	2 (20)	14 (20.3)	62 (9.8)
Nodule/mass	127 (13.7)	36 (16.7)	2 (20)	10 (14.5)	78 (12.4)
Pleural tick	239 (25.8)	68 (31.6)	6 (60)	18 (26.1)	147 (23.3)
Pleural effusion	68 (7.3)	16 (7.4)	0	4 (5.8)	48 (7.6)
Bronchiectasis	148 (16.0)	29 (13.5)	6 (60)	13 (18.8)	99 (15.7)
Extent†					
Minimal	584 (63.1)	83 (38.6)	4 (40.0)	44 (64.7)	451 (71.6)
Moderate	231 (24.9)	84 (39.1)	4 (40.0)	16 (23.5)	126 (20.0)
Advanced	111 (12.0)	48 (22.3)	2 (20.0)	8 (11.8)	53 (8.4)

*NTM, nontuberculous mycobacteria; TB, tuberculosis. †Minimal: lesions that are slight to moderate density or bronchiectasis without cavitation. Lesions may be present in a small portion of 1 or both lungs, but the total extent of the lesions should not exceed the volume of lung on 1 side, which is present above the second chondrosternal junction (or apex of 1 lung). Moderate: lesions may be present in 1 or both lungs, but the total extent must not be more than the following: 1) scattered lesions of slight to moderate density or bronchiectasis that may extend throughout the total volume of 1 lung or the equivalent volume in both lungs; 2) dense, confluent lesions that are limited in extend to one third of the volume of 1 lung; 3) cavitation with a diameter <4 cm. Advanced: lesions that are more extensive than moderately advanced.

Only 2 factors were associated with isolation of NTM: history of TB (adjusted OR 1.56, 95% CI 1.04–2.33) and HIV infection (adjusted OR 4.63, 95% CI 1.68–12.77) (Table 4). The most common NTM species were *M. fortuitum* and *M. abscessus* (rapid-growing mycobacteria) and *M. intracellulare*, *M. gordonae*, and *M. scrofulaceum* (slow-growing mycobacteria) (Table 5). Of 124 patients with NTM isolates (excluding 4 patients with both MTBc and NTM isolates), 14 (11.3%) had the same NTM species isolated in 2 separate sputum specimens. The same NTM species was isolated more than once during follow-up in 8/61 (13.1%) patients with symptomatic NTM colonization (3 *M. fortuitum*, *2 M. intracellulare*, 2 *M. abscessus*, and 1 *M. simiae*) versus 2/49 (4.1%) in patients with asymptomatic NTM colonization (2 *M. fortuitum*) (p = 0.009).

**Table 4:** Characteristics associated with the detection of NTM isolates in specimens of 1,179 presumptive pulmonary TB patients in a study of NTM infections at Kampong Cham Provincial Reference Hospital, Cambodia, October 1, 2012–April 21, 2014*

Characteristic	NTM isolate detected, no. (%)	Univariate analysis			Multivariate analysis	
		OR	95% CI		aOR	95% CI
Age group, y						
<40	24/295 (8.1)	1			1	
40–65	67/575 (11.6)	1.49	0.91–2.43		1.40	0.985–2.31
>65	33/309 (10.7)	1.35	0.78–2.34		1.33	0.76–2.35
Sex						
M	52/571 (9.1)	1			1	
F	72/608 (11.8)	1.34	0.92–1.95		1.36	0.93–2.00
Occupation						
Farmer	52/564 (9.2)	1				
Student, administration	17/138 (12.3)	1.38	0.77–2.48			
Factory, construction	3/63 (4.8)	0.49	0.15–1.62			
Fisherman	4/19 (21.0)	2.62	0.84–8.20			
Pensioner, unemployed	48/395 (12.1)	1.36	0.90–2.06			
Pneumoconiosis risk						
No	123/1,151 (10.7)	1				
Yes	1/27 (3.7)	0.31	0.04–2.39			
Drinking water						
Water well	70/704 (9.9)	1				
Running water	29/209 (13.9)	1.46	0.92–2.32			
Rain	7/63 (11.1)	1.13	0.50–2.58			
River/lake/pond	11/131 (9.2)	0.91	0.48–1.74			
Mineral water	6/72 (8.3)	0.82	0.34–1.97			
Water disinfection						
Boiled	73/696 (10.5)	1				
Filtered	15/121 (12.4)	1.21	0.67–2.18			
None	36/361 (10.0)	0.94	0.62–1.44			
Use of hot tub	38/331 (11.5)	1				
No	1/13 (7.7)	1				
Yes	85/835 (10.2)	0.64	0.08–5.01			
Unknown		0.87	0.58–1.31			
Smoke						
No	86/741 (11.6)	1				
Current smoker	12/173 (6.9)	0.57	0.30–1.06			
Former smoker	26/265 (9.8)	0.83	0.52–1.31			
TB history						
No	80/870 (9.2)	1			1	
Yes	44/307 (10.3)	1.65	1.11–2.45		1.56	1.04–2.33
HIV						
Negative	89/894 (10.0)	1			1	
Positive	6/19 (31.6)	4.17	1.55–11.25		4.63	1.68–12.77
Unknown	29/266 (10.9)	1.11	0.71–1.72			
Hospitalization for respiratory disease						
No	103/1,033 (10.0)	1				
Yes	21/144 (14.6)	1.54	0.93–2.55			
Health seeking behaviors						
No consultation	36/335 (10.7)	1				
Health center	21/234 (9.0)	0.81	0.46–1.44			
Private clinic	19/170 (11.2)	1.04	0.58–1.88			
Self-medication	5/36 (13.9)	1.34	0.49–3.66			
Unknown	43/404 (10.5)	0.99	0.62–1.58			

*aOR, adjusted OR; NTM, nontuberculous mycobacteria; OR, odds ratio; TB, tuberculosis.

**Table 5:** NTM species detected in a study of NTM infections at Kampong Cham Provincial Reference Hospital, Cambodia, October 1, 2012–April 21, 2014*

NTM species	No. (%) patients			
	Overall, N = 123	NTM disease, n = 11	NTM colonization, symptomatic, n = 61	NTM colonization, asymptomatic, n = 50
Slow-growing	56			
* M. avium*	2 (1.6)	1 (9.1)		1 (2.0)
* M. kansaii*	1 (0.8)			1 (2.0)
* M. scrofulaceum*	13 (10.5)	1 (9.1)	5 (8.2)	7 (14.3)
*M. intracellulare*†	23 (18.7)	5 (45.4)	12 (119.7)	6 (12.0)
* M. gordonae*	13 (10.5)		8 (13.1)	5 (10.2)
* M. interjectum*	1 (0.8)		1 (1.6)	
* M. simiae*	3 (2.4)	1 (9.1)	2 (3.3)	
Rapid-growing	45			
* M. fortuitum*	28 (22.3)	1 (9.1)	16 (26.2)	11 (22.0)
* M. abscessus*	14 (11.3)	2 (18.2)	6 (9.8)	6 (12.2)
* M. asiaticum*	2 (1.6)		1 (1.6)	
*M abscessus* and *M. asiaticum*	1 (0.8)		1 (1.6)	
Not identified	22 (17.7)		9 (14.7)	13 (26.5)

*NTM, nontuberculous mycobacteria.  †In 3 cases, *M. intracellulare* was recovered with another NTM species: *M. scrofulaceum* (n = 1), *M. interjectum* (n = 1), and *M. fortuitum* (n = 1).

Overall, 217/1183 (18.3%) patients were culture-positive for MTBc, including 142/942 (15.1%) by LJ and 204/1134 (18.0%) by MGIT. The NTM growth was 2.7% (32/1170) on LJ medium compared with 8.0% (95/1,186) in MGIT (p<0.001). Overall NTM growth was 10.0% (117/1,187) when methods were combined.

Of 1,183 patients, 197 were smear-positive (16.6%). Of these, 171 (86.8%) were confirmed to have MTBc (167 by culture and 4 by XpertMTB/RIF only), 11 (5.6%) were positive for NTM, and 15 (7.6%) were both culture and XpertMTB/RIF negative. XpertMTB/RIF detected MTBc in 166/197 (84.2%) smear-positive patients. After exclusion of contaminated results, LJ and MGIT were positive for MTBc in 122/157 (77.7%) and 162/195 (83.1%) of smear-positive patients and in 20/785 (2.5%) and 42/942 (4.4%) of smear-negative patients, respectively.

Out of 128 patients with NTM isolates (including 4 patients with both MTBc and NTM isolates), 12 met the ATS criteria for having NTM lung disease but 4 (33.3%) were finally not confirmed by the study expert committee because of the good clinical response to an antimicrobial drug treatment targeting bacterial respiratory infection (1 for *Klebsiella pneumonia*, 1 for *Pseudomonas aeruginosa*, and 2 empirical treatments). No patient had clinical exacerbation or NTM recurrence during the 12 months of follow-up. Of the 11 patients diagnosed with NTM lung disease by the expert committee, 2 (18.2%) did not meet all the ATS criteria. Both were advanced HIV-infected patients; 1 was symptomatic with normal chest x-ray with only 1 *M. simiae* isolate, and 1 had a nonsuggestive chest radiograph results and several *M. intracellulare* isolates from different specimens (Table 6).

**Table 6:** Comparison of American Thoracic Society classification criteria used to define NTM disease with those used in a study of NTM infections at Kampong Cham Provincial Reference Hospital, Cambodia, October 1, 2012–April 21, 2014*

Radiologic criteria	Clinical criteria, in addition to cough of >3 wks duration			
	Same NTM species in 2 sputum samples‡	NTM in only 1 sputum sample	NTM and MTBc in 1 sputum sample	Total
Chest radiograph results				
Suggestive†‡	12 (8)‡	24 (1)	4	40
Abnormal but nonsuggestive	2 (1)	44	0	46
Normal	3	39 (1)	0	42
Total	16	108	4	128

*CR, chest radiograph; MTBc, *Mycobacterium tuberculosis* complex; NTM, nontuberculous mycobacterial.  †Presence of nodules, cavities, or bronchiectasis. ‡Criteria corresponding to the NTM lung disease classification according to the American Thoracic Society. Numbers in parentheses correspond to the cases classified as NTM lung disease by the study’s expert committee.

## Discussion

We report a high prevalence (10.8%) of NTM isolates in sputum specimens of patients with presumptive TB. NTM growth occurred in one third of patients with positive mycobacterial cultures. However, only 10% of them were diagnosed with NTM disease. The prevalence of NTM isolates was higher than what has been previously reported in Cambodia among HIV infected patients (1%) (*27*). However, at that time, only LJ culture was used, which is likely to explain the difference with our findings. In our study, the NTM growth in MGIT was more than twice as high (8.0%) than on LJ media (2.7%), and LJ missed 2 out the 11 cases finally diagnosed as NTM disease; these findings support the ATS recommendation to use both solid and liquid culture methods for diagnosis of NTM disease (*1*). Also, in the same survey among HIV-infected patients, the prevalence of NTM isolates was 30% and 7%, respectively, in Thailand and Vietnam sites where MGIT was also used (*27*). Our findings are similar to the results from another study in Zambia, where 11% of patients with chronic cough had NTM isolates in their sputum and 0.6% had NTM disease (*16*).

The proportion of NTM disease cases among patients with NTM isolates in our study (10%) is at the lower bound of the range of 9%–71% reported in a systematic review of hospital-based studies in Southeast Asia and is lower than has been reported (30%) in the survey among HIV-infected outpatients in Thailand and Vietnam (*27*,*28*). The prospective follow-up of patients with NTM isolates in our study could partially explain the low proportion of disease. Indeed, retrospective hospital-based surveys tend to overestimate the prevalence because of recruitment bias and small numbers. By using a cross-sectional design only, we might have classified 4 additional cases as NTM disease because the patients met the ATS criteria at enrollment. However, their clinical assessment during follow-up showed that their cases qualified better as active or transient NTM colonization rather than NTM disease (*4*,*11*).

In our setting, two thirds of patients with NTM lung disease had fibrocavitary disease, with bronchiectasis in 60 and cavititation in 40% (*11*). Post-TB inflammatory bronchiectasis was the most common underlying structural lung disease. However, discussion persists regarding whether NTM invade preexisting bronchiectasis or lead to the initiation of bronchiectasis through postinflammatory bronchiectasis like MTBc does (*29*,*30*). Another possibility is that some NTM disease might previously have been mistakenly attributed to MTBc. One third of patients in our study had advanced HIV infection without underlying lung disease and had disseminated NTM infection with some lung involvement. This finding can explain why only half of them had illness that met the ATS criteria (*3*,*20*).

Among the 113 patients classified as having NTM colonization, the group of symptomatic patients were older and had a history of TB or hospitalization for respiratory disease and bronchiectasis more often than did the group of asymptomatic patients. Also, recurrence of the same NTM species in a sputum specimen during follow-up was more common among symptomatic patients than asymptomatic patients (13.1% vs. 4.1%). Based on these criteria, patients with NTM isolates no longer symptomatic at first follow-up visit were more likely to have NTM contamination or transient colonization without progression, whereas patients who remained symptomatic were more likely to have active colonization or indolent infection, which can progress to active (overt) disease (*4*,*11*,*20*).

Rapid-growing NTM species were very commonly found in our study. The proportion of *M. fortuitum* isolates (28.7%) was much higher than what has been previously reported in surveys conducted in Asia (*31*). However, as expected, *M. fortuitum* was only occasionally responsible for NTM lung disease (*2*). The proportion of *M. abscessus* isolates (14.8%) was close to what has been reported in other studies in Taiwan (18%) and China (14%) and was responsible for 18% (2/11) of the NTM lung disease cases in our study (*28*). This fact is particularly concerning because *M. abscessus* is usually resistant to antimicrobial drugs (*11*). The prevalence of isolation of *M. intracellulare* (23.8%) and *M. scofulaceum* (13.9%) was also very close to what has been reported in previous surveys in Asia (*28*,*32*). Surprisingly, *M. kansasii*, which is frequently isolated in specimens from patients in Asia, was almost absent in our setting (*28*).

If we apply our results to other settings in Cambodia without availability of mycobacterial culture, 10% of patients with presumptive TB (NTM culture-positive) could be wrongly started on TB treatment. Of these patients, those with smear-positive TB would definitely be started on TB treatment. Also, smear-negative patients classified as having NTM lung disease or symptomatic NTM colonization (55/986 [5.6%]) would probably also have been started on TB treatment. Even if NTM isolates represented a small proportion of all smear-positive cases (5%), in settings with high NTM disease burden, confirmation of smear-positive TB using XpertMTB/RIF should be considered. This practice might pose a challenge for national TB programs in countries with low HIV prevalence where XpertMTB/RIF is used as add-on test for smear-negative TB patients (*19*,*31*). Clinicians should also take into consideration the possibility of NTM infection in patients not responding to TB treatment, smear-negative patients with recurrent respiratory infections with bronchiectasis, patients with post-TB lung damage, and HIV-infected patients. Mycobacterial culture should be requested in such cases.

The only factors associated with NTM isolates in patients’ specimens were history of previous TB and HIV infection. Disorders of cell-mediated immunity are known to be associated with an increased risk for disseminated NTM disease (*3*,*15*). However, to our knowledge, HIV has not yet been identified as a risk factor for a positive NTM culture from respiratory specimens (*16*,*33*,*34*).

Our study has limitations. First, even though the prospective design is a strength of the study, 12 months might be too short a duration to assess the progression to NTM lung disease in patients with symptomatic NTM colonization. On the other hand, using findings from patients’ follow-up for final classification diverged from the ATS criteria and might have underestimated the proportion of NTM lung disease in our study compared with previous studies. Indeed, 4 out of 12 patients who fit the ATS criteria for lung disease at initial assessment were not confirmed by the study expert review committee. However, this potential underreporting is offset by the fact that 3 patients whose cases did not meet the ATS criteria were classified as having NTM lung disease. Second, assessing the evolution from NTM colonization to disease in patients who dropped out during follow-up (19.3%) was not possible. Third, properly assessing the presence of bronchiectasis in the study population in the absence of computed tomography scan was difficult. Last, the survey included patients with presumptive TB in a limited rural area in Cambodia, so the findings might not be generalizable to other populations.

In conclusion, our study shows that the prevalence of NTM isolates can be high among patients with presumptive TB, which can result in potential misdiagnosis and treatment of TB. In settings without routine use of mycobacterial culture, a sentinel survey of the NTM disease prevalence among patients with clinical suspicion of TB might be recommended. Then, in areas where NTM isolates are frequently detected, such as Southeast Asia, clinicians should consider systematic confirmation of TB in smear-positive patients with XpertMTB/RIF assay and assessment of potential NTM disease in smear-negative patients not responding to TB treatment or in patients with recurrence of respiratory symptoms and structural lung disease. This approach requires specific training to build expertise, good radiography services, and improved access to mycobacterial culture and NTM speciation. Our study highlights the limitations of the 2007 ATS criteria, especially for HIV-infected patients and patients with multiple respiratory infections. Our findings reflect the complex interaction between the NTM exposure–related factors and host-related factors resulting in a spectrum of conditions between colonization and actual disease that can be difficult to differentiate (*4*).
